# Evaluation of Tissue Adequacy by Computed Tomography-Guided Needle Biopsy for Comprehensive Genomic Profiling Following Chemotherapy

**DOI:** 10.7759/cureus.109141

**Published:** 2026-05-18

**Authors:** Kazuya Kawashima, Kenichi Kato, Tomohiro Suzuki, Misato Sone, Makoto Hamano, Shigekatsu Maekawa, Mitsumasa Osakabe, Kunihiro Yoshioka

**Affiliations:** 1 Department of Radiology, Iwate Medical University School of Medicine, Yahaba, JPN; 2 Department of Radiology, Hachinohe Red Cross Hospital, Hachinohe, JPN; 3 Department of Urology, Iwate Medical University School of Medicine, Yahaba, JPN; 4 Department of Molecular Diagnostic Pathology, Iwate Medical University School of Medicine, Yahaba, JPN

**Keywords:** chemotherapy, comprehensive genomic profiling, ct-guided biopsy, fdg pet/ct, interventional radiology, next-generation sequencing, tissue adequacy

## Abstract

Background

Computed tomography-guided needle biopsy (CT-NB) has become increasingly important for comprehensive genomic profiling (CGP). However, factors influencing tissue adequacy for CGP after chemotherapy remain unclear.

Purpose

This study aimed to evaluate the factors affecting the tissue adequacy of CT-NB specimens for CGP following chemotherapy.

Materials and methods

We retrospectively reviewed patients who underwent CT-NB for intended CGP after chemotherapy between June 2020 and December 2024. Tissue adequacy was defined as tumor content ≥20% on pathological assessment. Adequacy rates were calculated, and associations with tumor size, number of needle passes, and 18F-fluorodeoxyglucose (FDG) uptake were evaluated.

Results

This study included 33 patients (median age: 71 years). Biopsy sites were the bone (n=9), liver (n=8), lymph nodes (n=5), and others (n=11). One procedure was technically unsuccessful, and five patients did not proceed to CGP owing to clinical deterioration or other reasons. Among 27 evaluable patients, 17 (63%) provided adequate specimens, whereas 10 (37%) were inadequate owing to insufficient tumor cells. Tissue adequacy was not significantly associated with tumor size or the number of needle passes. Among FDG uptake parameters (n=16), the maximum standard uptake value (SUVmax) showed no significant difference between the adequate and inadequate groups, whereas the mean standard uptake value (SUVmean) demonstrated a statistically significant difference between both groups. Ultimately, the SUVmax/SUVmean ratio showed no significant relationship with tissue adequacy.

Conclusion

CT-NB can provide tissue for CGP after chemotherapy; however, adequate tissue acquisition remains challenging. In patients with available FDG-PET/CT, a higher SUVmean was associated with tissue adequacy.

## Introduction

Computed tomography-guided needle biopsy (CT-NB) is an established procedure for obtaining histopathological diagnosis in patients with cancer [1]. In addition to histopathological evaluation, CT-NB is also used to confirm tumor biomarker status for molecular targeted therapy. With advances in personalized medicine, cancer treatment now relies on an individual's genomic information. Consequently, the biopsy tissue samples must be of sufficient quality for genomic analysis [2-5].

In Japan, comprehensive genomic profiling (CGP) was conducted by the National Health Insurance in June 2019. CGP requires tissue specimens with a high tumor content, which are typically obtained via surgery or biopsy. Although surgical specimens can provide sufficient tissue volume, they only reflect genetic changes at the time of surgery. In contrast, biopsy specimens may more accurately reflect the genetic status of recurrent tumors. Therefore, opportunities for CT-NB in CGP have increased.

The utility of CT-NB for CGP has been reported mainly in lung tumors [6-10], whereas its effectiveness in other tumors, such as genitourinary tumors, remains insufficiently investigated. Furthermore, treatment-naïve specimens are considered advantageous for CGP analysis [11]. However, in Japan, CGP is primarily administered to patients with recurrent disease or tumors showing insufficient response to standard chemotherapy, after completion or anticipated completion of standard treatment. This study aimed to evaluate the adequacy of biopsy specimens obtained via CT-NB after chemotherapy and identify the factors that influence specimen adequacy.

## Materials and methods

Patient selection

We retrospectively reviewed our institutional radiology database to identify consecutive patients.The inclusion criteria were as follows: (i) patients who underwent CT-NB between June 2020 and December 2024 and (ii) biopsy was performed for CGP following standard chemotherapy or prior to its anticipated completion. The exclusion criteria were as follows: (i) fluid aspiration biopsy for cell block technique without targeted solid tumor and (ii) patients for whom CGP analysis was newly proposed after the biopsy procedure. This single-center study was conducted at Iwate Medical University Hospital in Yahaba, Iwate, Japan, and approved by the Institutional Review Board of Iwate Medical University (approval number: MH2024-151; approval date: March 14, 2025), and written informed consent for biopsy for CGP was obtained from all patients. 

Indication of CGP in Japan

In Japan, CGP is reimbursed under the national health insurance system for patients with solid malignancies who meet one of the following criteria: (1) completion or expected completion of standard therapy for advanced or recurrent disease or (2) cancer of unknown primary origin. CGP testing was performed at designated cancer genomic medicine core hospitals or equivalent institutions, and all cases were reviewed by an expert panel. All patients included in this study met these insurance coverage criteria. All positron emission tomography (PET)/CT scans were performed using Discovery IQ PET/CT (GE Healthcare, Chicago, Illinois, United States) approximately 60 minutes after intravenous injection of 2-5 MBq/kg of 18F-fluorodeoxyglucose (FDG).

Biopsy procedure

All biopsies were performed by, or under the supervision of, board-certified interventional radiologists, who were informed in advance of the intended CGP analysis. Target lesions were selected based on CT, and FDG-PET/CT was additionally used, when available, to preferentially sample FDG-avid regions.

Biopsies were performed under local anesthesia with CT guidance (Aquilion Prime SP; Canon Medical Systems Corporation, Otawara, Japan). Intermittent, operator-controlled CT fluoroscopy was used to advance the biopsy needle. Initially, an 18-G side-notch needle (Super Core®, Argon Medical Devices, Plano, Texas, United States) without a coaxial system was used; subsequently, an 18-G full-core needle (CorVocet®, Merit Medical Systems, South Jordan, Utah, United States) with a coaxial system became standard. For sclerotic bone lesions, an 11-G trephine needle (T-LOK®, Argon Medical Devices, Plano, Texas, United States) was employed. The attending radiologist determined the number of cores to maximize tissue acquisition for both pathological and genomic analyses. On-site cytopathology was performed in all cases to confirm the presence of tumor cells.

Tissue requirements for CGP

The CGP platform used was FoundationOne CDx (Foundation Medicine, Inc., Cambridge, Massachusetts, United States), which analyzed 324 genes in formalin-fixed paraffin-embedded specimens. Required sample criteria included (1) tumor cell content ≥20% (≥30% recommended) and (2) tissue equivalent to at least 10 5-μm unstained slides (≥20 recommended). The decision to submit specimens for CGP was made by institutional pathologists.

Outcome measures

The primary outcome was tissue adequacy for CGP using CT-NB. Factors potentially associated with tissue adequacy for CGP, including tumor size, number of punctures, and FDG uptake, were also evaluated. The technical success of the biopsy was defined as the successful placement of the biopsy needle within the target lesion and acquisition of tissue for histopathological evaluation. Tumor size was defined as the maximum diameter of the target lesion at the biopsy site on cross-sectional imaging. The number of punctures was defined as the number of sampling procedures performed using a coaxial system. FDG uptake was assessed in patients who underwent FDG-PET/CT at our hospital prior to CT-NB. The maximum standard uptake value (SUVmax) was measured using a dedicated viewer system (EV Insite.R®, PSP Corporation, Tokyo, Japan). A two-dimensional region of interest corresponding to the biopsy site was analyzed. In addition, the mean standard uptake value (SUV mean) and SUVmax/mean standard uptake value (SUVmean) ratio were evaluated as indicators of intralesional metabolic heterogeneity.

Statistical analysis

Associations between adequate or inadequate specimens and clinical factors were analyzed using the Mann-Whitney U test. For metabolic parameters (SUVmax, SUVmean, and SUVmax/SUV mean ratio), comparisons between the adequate and inadequate groups were also performed using the Mann-Whitney U test. All analyses were two-sided, with significance set at p<0.05. Analyses were conducted using Statcel-the Useful Addin Forms on Excel, 5th edition (OMS Publishing Inc., Saitama, Japan).

## Results

Patient characteristics

In total, 33 patients underwent CT-NB for intended CGP analysis during the study period. The median age was 71 years (range: 29-93 years), and prostate cancer was the most frequent diagnosis (51.5%). Patient characteristics are summarized in Table 1.

**Table 1:** Patient characteristics

Variable	Value
Gender (male/female)	30 (90.9%)/3 (9.1%)
Age, median (IQR), years	71 (64-77)
Clinical diagnosis (primary site)	-
Prostate cancer	17 (51.5%)
Penile cancer	2 (6.1%)
Renal cancer	2 (6.1%)
Testicular tumor	2 (6.1%)
Thymic cancer	2 (6.1%)
Urachal cancer	2 (6.1%)
Ureteral cancer	2 (6.1%)
Others	4 (12.1%)

Major biopsy sites were the bone (27.3%) and liver (24.2%). The most used device was the coaxial full-core biopsy needle (67.6%), although multiple needle types were used in some patients. Additional details are presented in Table 2.

**Table 2:** Biopsy site and needle *In one patient, the coaxial full-core biopsy needle and side-notch needle were used in duplicate.

Biopsy site	n=33	%
Bone	9	27.3%
Liver	8	24.2%
Lymph node	5	15.2%
Pelvic cavity	4	12.1%
Retroperitoneum	2	6.1%
Lung	2	6.1%
Other	3	9.1%
Biopsy needle	n=34*	%
Coaxial full-core biopsy needle	23	67.6%
Side-notch needle	6	17.6%
Trephine needle	5	14.7%

Tissue adequacy for CGP

**Figure 1:**
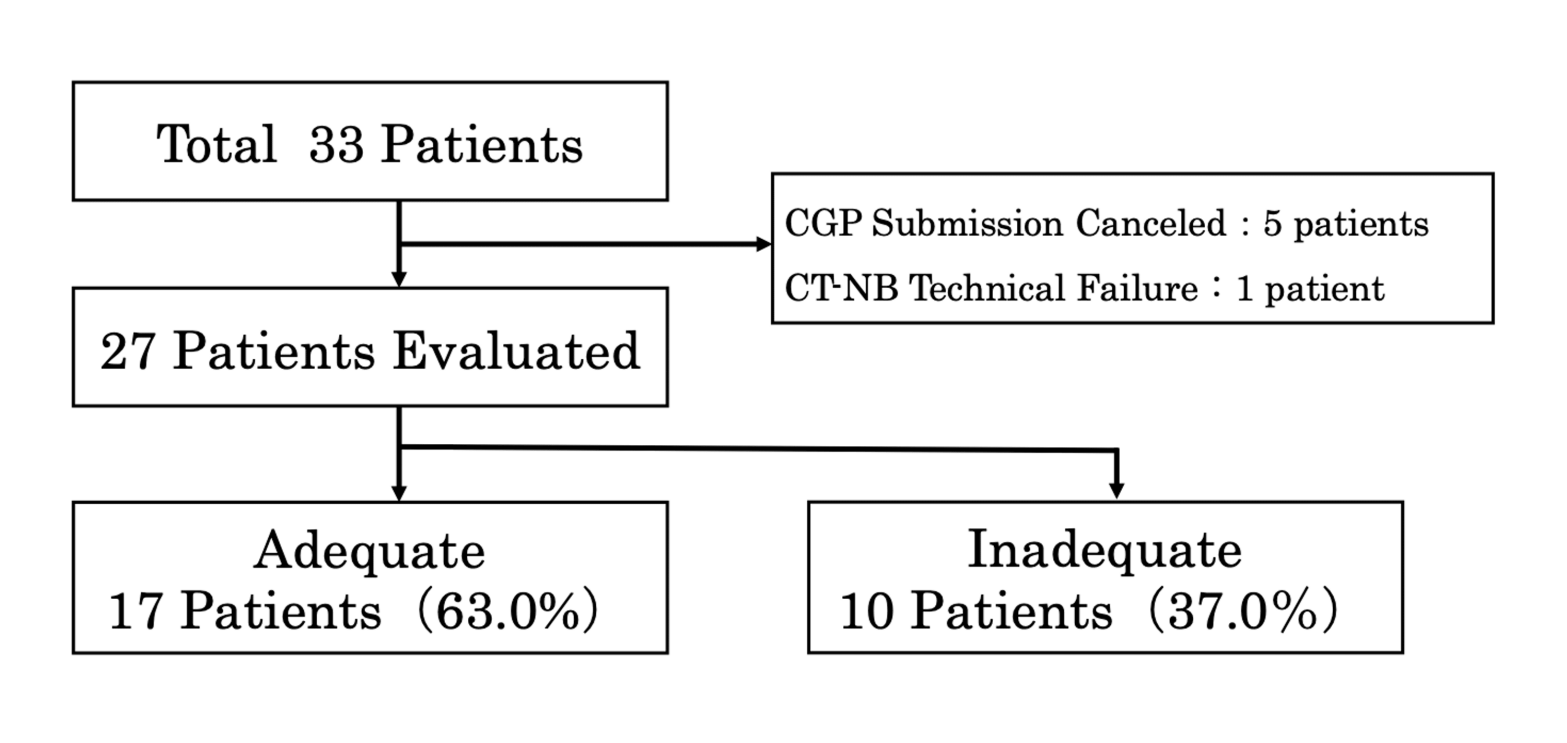
Flow diagram of patient inclusion and tissue adequacy for CGP CGP: comprehensive genomic profiling; CT-NB: computed tomography-guided needle biopsy

Excluding one technical failure, histopathological diagnoses were obtained in 32 patients. Of these, five did not proceed to CGP (four due to clinical deterioration and transition to best supportive care and one due to financial reasons). Ultimately, CGP was performed in 27 patients (Figure 1).

Adequate tissue for CGP submission was achieved in 17 patients (63%), whereas 10 patients (37%) were deemed inadequate because of insufficient tumor cells.

Correlation of tissue adequacy with size, needle punctures, and FDG uptake

The associations between tissue adequacy for CGP and tumor size, number of punctures, and degree of FDG uptake were evaluated (Table 3).

**Table 3:** Comparison of tissue adequacy for CGP ^†^Mann-Whitney U test CGP: comprehensive genomic profiling; SUVmax: maximum standard uptake value; SUVmean: mean standard uptake value

Variable	Adequate for CGP (n)	Inadequate for CGP (n)	Test statistic	P-value^†^
Tumor size (mm)	56 (25-76) (n=17)	38.5 (23.8-54.5) (n=10)	U=58	0.32
Number of needle passes	7.5 (5-9) (n=10)	6.5 (5-8) (n=10)	U=44	0.41
SUVmax	7.74 (5.56-11.47) (n=9)	5.89 (4.52-6.70) (n=7)	U=20	0.12
SUVmean	5.32 (4.12-7.9) (n=9)	3.35 (2.55-4.11) (n=7)	U=12	0.01
SUVmax/SUVmean	1.45 (1.29-1.57) (n=9)	1.55 (1.48-1.72) (n=7)	U=28	0.22

**Figure 2:**
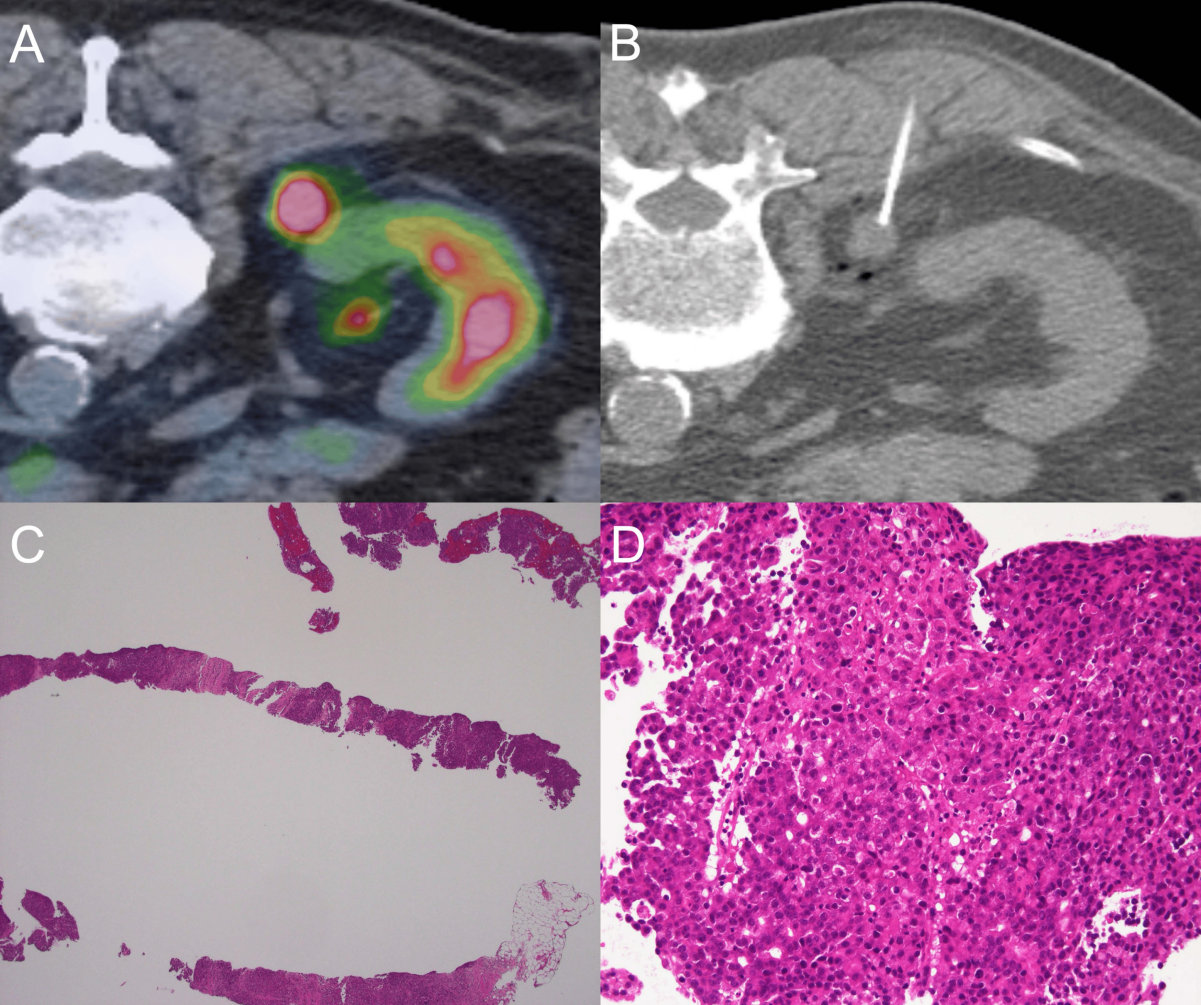
A 77-year-old man with treatment-resistant prostate cancer (A) FDG-PET/CT demonstrates abnormal accumulation in the right perirenal tumor. The SUVmax, SUVmean, and SUVmax/SUVmean ratio are 5.56, 3.55, and 1.57, respectively. (B) CT fluoroscopy image during CT-guided biopsy. The perirenal tumor was punctured using a coaxial biopsy system. Seven core samples were obtained. (C) Low-power view of H&E-stained sections shows abundant tumor cells with >80% content. (D) High-power view of the H&E section demonstrates proliferation of tumor cells suitable for CGP testing. FDG: 18F-fluorodeoxyglucose; PET: positron emission tomography; CT: computed tomography; SUVmax: maximum standard uptake value; SUVmean: mean standard uptake value; H&E: hematoxylin and eosin; CGP: comprehensive genomic profiling

**Figure 3:**
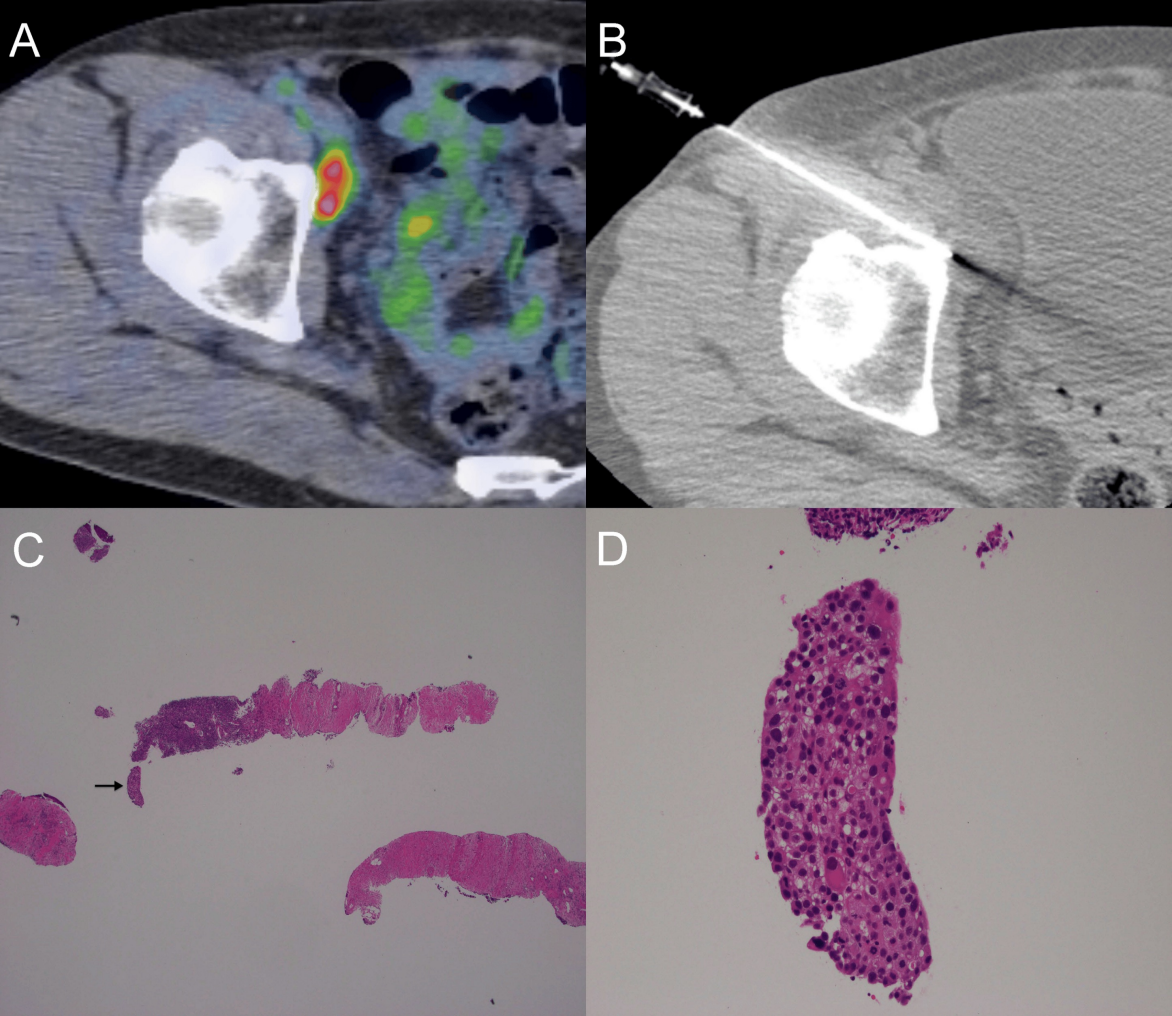
A 66-year-old man with right ureteral carcinoma and multiple lymph node and lung metastases. The lymph node progressed despite second-line chemotherapy (A) FDG-PET/CT demonstrates an FDG-avid lesion along the right iliac artery. The SUVmax, SUVmean, and SUVmax/SUVmean ratio were 5.98, 4.01, and 1.49, respectively. (B) CT fluoroscopy image during CT-guided biopsy shows a coaxial needle inserted near the lymph node. (C) Low-power view of H&E-stained sections shows predominantly inflammatory cells and fibrous tissues. Tumors (→) are only observed in a very small portion. (D) A small number of tumor cells suggestive of ureteral carcinoma were observed in high-power field images of H&E-stained sections. FDG: 18F-fluorodeoxyglucose; PET: positron emission tomography; CT: computed tomography; SUVmax: maximum standard uptake value; SUVmean: mean standard uptake value; H&E: hematoxylin and eosin

Regarding tumor size and number of needle punctures with the coaxial needle system, no significant difference was noted between adequate and inadequate tissues for CGP. FDG-PET/CT was performed in 16 patients, with a median of 28 days before CT-NB. Regarding FDG accumulation, the median SUVmax in adequate and inadequate tissues for CGP was 7.74 and 5.89 (p=0.12), respectively. In addition, the median of SUVmean in adequate and inadequate tissue was 5.32 and 3.35 (p=0.01), respectively. The median SUVmax/SUVmean ratio was 1.45 and 1.55 (p=0.22) in adequate and inadequate specimens, respectively. Among these FDG metabolic parameters, SUVmean showed a significant difference between the two groups. Figures 2-3 show representative cases of adequate and inadequate tissues, respectively.

The reasons for the inadequacy despite FDG accumulation were the presence of inflammatory cells and insufficient tumor content on pathological analysis.

## Discussion

Recent advances in precision oncology have increased the demand for image-guided biopsies that can provide sufficient tissue for genomic profiling [10]. The usefulness of CT-NB in CGP has been studied mainly in lung cancer, with reported success rates ranging from 53.2% to 91% [6-10]. However, data on percutaneous biopsy for CGP in other malignancies remain limited. In this study, we observed a 63% adequacy rate in a cohort largely composed of patients with urogenital tumors.

Several factors that may affect tissue adequacy include tumor size, biopsy needle, tumor characteristics, imaging guidance, and the presence or absence of chemotherapy or radiotherapy [4,7,11]. Previous reports on tumor size and adequacy of biopsy have been inconsistent. Some reports, similar to our study, suggest that tumor size is not a factor [4,12], whereas others report that DNA collection may be insufficient for tumors <1 cm [7,13]. On the other hand, some studies report that tumors >3 cm may also result in inadequate specimens due to necrosis [11]. These results emphasize the importance of targeting viable tumor tissues rather than relying solely on the tumor size.

Although larger needles can yield more tissue, 18-G needles were used for all nonbone lesions in our series, with coaxial systems preferred in later cases. The number of biopsies performed using the coaxial system was determined by the interventional radiologist; however, as a general rule, ≥5 cores were obtained, and no difference was observed in the number of cores between specimens adequate and inadequate for CGP.

In CT-NB for CGP, tissues must be collected from viable, tumor cell-rich areas [14]. When available as an imaging reference, FDG-PET/CT was used to guide biopsies to FDG-avid lesions. However, in this study, no significant difference in SUVmax, the indicator representing maximum FDG accumulation, was observed between specimens adequate and inadequate for CGP. We hypothesized that intratumoral heterogeneity may account for the difference between adequate and inadequate specimens for CGP. Consequently, we evaluated intratumoral heterogeneity by assessing the SUVmean and SUVmax/SUVmean ratio [15,16]. Although the SUVmean reflected tumor heterogeneity associated with necrosis or granuloma, the SUVmax/SUVmean ratio showed no significant differences between the two groups. In Japan, CGP is limited to patients who have completed standard chemotherapy, which may increase the proportion of non-tumorous cells in specimens [11]. Although FDG uptake can reflect both tumor cells and inflammation or granulomatous reactions [17], distinguishing between tumor-related accumulation and inflammatory accumulation prior to biopsy remains challenging.

This study has certain limitations. First, its retrospective, single-center design inevitably introduced selection bias and limits the generalizability of the findings. Second, the relatively small sample size reduced the statistical power and may have contributed to the lack of significant associations between tissue adequacy and clinical or imaging factors. Third, although biopsies were performed by experienced interventional radiologists, technical variability, including needle type, number of punctures, and operator discretion, may have influenced outcomes. Fourth, the pathological assessment of tumor content was performed based on institutional standards, which may not be fully standardized across institutions. Finally, since CGP testing in Japan is restricted to patients who have completed standard therapies, the study population may inherently have a higher proportion of necrotic or fibrotic tissue, potentially underestimating adequacy rates for CGP compared to treatment-naïve cohorts.

## Conclusions

This retrospective study demonstrates that the success rate of obtaining tissue for CGP via CT-NB in patients after chemotherapy was 63%. Furthermore, our study suggests that tissue sampling from lesions with higher SUVmean on FDG-PET plays a role in successful genomic profiling. However, tissue adequacy may be influenced by various factors. Further studies are needed to identify factors associated with successful sampling and to improve biopsy target selection.
